# SARS-CoV-2 N protein induced acute kidney injury in diabetic db/db mice is associated with a Mincle-dependent M1 macrophage activation

**DOI:** 10.3389/fimmu.2023.1264447

**Published:** 2023-11-03

**Authors:** Wenjing Wu, Wenbiao Wang, Liying Liang, Junzhe Chen, Sifan Sun, Biao Wei, Yu Zhong, Xiao-Ru Huang, Jian Liu, Xiaoqin Wang, Xueqing Yu, Hui-Yao Lan

**Affiliations:** ^1^ Guangdong Cardiovascular Institute, Guangzhou, China; ^2^ Guangdong-Hong Kong Joint Laboratory for Immunological and Genetic Kidney Disease, Departments of Nephrology and Pathology, Guangdong Provincial People’s Hospital (Guangdong Academy of Medical Sciences), Southern Medical University, Guangzhou, China; ^3^ Departments of Medicine & Therapeutics, Li Ka Shing Institute of Health Sciences, Lui Che Woo Institute of Innovative Medicine, The Chinese University of Hong Kong, Hong Kong, China; ^4^ The First Clinical College, Hubei University of Chinese Medicine, Wuhan, China; ^5^ Department of Nephrology, Hubei Provincial Hospital of Traditional Chinese Medicine, Hubei Province Academy of Traditional Chinese Medicine, Wuhan, China; ^6^ Department of Clinical Pharmacy, Guangzhou Eighth People’s Hospital, Guangzhou Medical University, Guangzhou, China; ^7^ Department of Nephrology, The Third Affiliated Hospital, Southern Medical University, Guangzhou, China; ^8^ Department of Nephrology, Jiangsu Province Hospital of Chinese Medicine, Affiliated Hospital of Nanjing University of Chinese Medicine, Nanjing, Jiangsu, China; ^9^ Department of Nephrology, The Affiliated Traditional Chinese Medicine Hospital of Southwest Medical University, Luzhou, China

**Keywords:** SARS-CoV-2, N protein, AKI, quercetin, Mincle, M1 macrophage

## Abstract

“Cytokine storm” is common in critically ill COVID-19 patients, however, mechanisms remain largely unknown. Here, we reported that overexpression of SARS-CoV-2 N protein in diabetic db/db mice significantly increased tubular death and the release of HMGB1, one of the damage-associated molecular patterns (DAMPs), to trigger M1 proinflammatory macrophage activation and production of IL-6, TNF-α, and MCP-1 via a Mincle-Syk/NF-κB-dependent mechanism. This was further confirmed *in vitro* that overexpression of SARS-CoV-2 N protein caused the release of HMGB1 from injured tubular cells under high AGE conditions, which resulted in M1 macrophage activation and production of proinflammatory cytokines via a Mincle-Syk/NF-κB-dependent mechanism. This was further evidenced by specifically silencing macrophage Mincle to block HMGB1-induced M1 macrophage activation and production of IL-6, TNF-α, and MCP-1 *in vitro*. Importantly, we also uncovered that treatment with quercetin largely improved SARS-CoV-2 N protein-induced AKI in db/db mice. Mechanistically, we found that quercetin treatment significantly inhibited the release of a DAMP molecule HMGB1 and inactivated M1 pro-inflammatory macrophage while promoting reparative M2 macrophage responses by suppressing Mincle-Syk/NF-κB signaling *in vivo* and *in vitro*. In conclusion, SARS-CoV-2 N protein-induced AKI in db/db mice is associated with Mincle-dependent M1 macrophage activation. Inhibition of this pathway may be a mechanism through which quercetin inhibits COVID-19-associated AKI.

## Introduction

Acute kidney injury (AKI) has been recognized as a common complication of the coronavirus disease 2019(COVID-19), which is caused by severe acute respiratory syndrome coronavirus 2 (SARS-CoV-2). AKI is common in critically ill COVID-19 patients with high mortality (1–4). Patients with older age and underlying diseases such as hypertension, diabetes, and chronic kidney disease (CKD) are at high risk for COVID-19 AKI (5, 6). However, mechanisms for COVID-19 AKI remain largely unknown.

Increasing evidence shows that cytokine storm is common in critically ill patients with AKI (7). Cytokine storm is related to excessive immune responses in patients with severe SARS-CoV-2 infection and is characterized by the production of large amounts of cytokines such as interleukin-6 (IL-6), interleukin-8 (IL-8), tumor necrosis factor-alpha (TNF-a), and monocyte chemotactic protein-1 (MCP-1) (8–13). Thus, understanding the mechanisms that lead to the cytokine storm associated with COVID-19 infection is extremely important for developing potential treatments for critically ill COVID-19 patients.

Elevated inflammatory markers such as white blood cell count, monocyte count, high levels of C-reactive protein, and proinflammatory cytokines such as IL-6, TNF-a, and MCP-1 have been demonstrated in patients with severe COVID-19 (10–15). It is also reported that the CD68^+^ macrophages infiltrating the kidney are also associated with severe kidney injury in COVID-19 patients (16). Tubular necrosis is a pathological feature of COVID-19 patients with AKI (7). Tubular necrosis can induce renal inflammation by releasing the damage-associated molecular patterns (DAMPs) to activate immune cells through identical pattern recognition receptors (PRR) (17). Macrophage-inducible C-type lectin (Mincle) is a transmembrane pattern recognition receptor that is expressed by M1 pro-inflammatory macrophages in response DAMPs (18, 19). Mincle acts via its downstream Syk and NF-κB signaling to activate M1 pro-inflammatory macrophages and is essential for maintaining the pro-inflammatory phenotype of M1 macrophages in AKI (20). It has been reported that increased expression of DAMPs such as the high-mobility group box 1 protein (HMGB1) and S100A8/A9 is found in patients with moderate to severe COVID-19 (21, 22). Early post-mortem examination confirms that SARS-CoV-2 can directly infect human kidney tubular cells and then induce acute tubular damage by a direct cytopathic effect and CD68-positive macrophages (23). Our recent study also finds that SARS-CoV-2 N protein can activate transforming growth factor beta (TGF-β) signaling by interacting with Smad3 and thus causes tubular cell death via mechanisms associated with Smad3-dependent G1 cell cycle arrest and Ripk3/MLKL necroptosis pathways (24–26). However, Mincle-dependent M1 macrophage activation in COVID-19 AKI remains unexplored, which was investigated in the present study.

Quercetin is a widespread flavonoid found in a large variety of Chinese herbs and dietary supplements. It shows multiple pharmacological effects, including antiviral, antioxidant, and anti-inflammatory properties. Clinical trials demonstrate that treatment of COVID-19 patients with oral quercetin significantly improves the severity of COVID-19 syndromes (27, 28). Molecular docking studies predict that quercetin can bind to multiple SARS-CoV-2 proteases and thus inhibit viral infection (29, 30). Our recent studies confirmed that ultrasound-microbubble-mediated kidney-specifically overexpressing SARS-CoV-2 N protein can induce AKI in 8-week-old db/db mice by causing tubular necrosis and elevated serum levels of creatinine and blood urea nitrogen, which is further exacerbated in older age (16 weeks) of db/db mice, but is inhibited by treatment with quercetin (24). We find that quercetin can effectively block the binding of SARS-CoV-2 N protein to Smad3, therefore inhibiting SARS-CoV-2 N protein-induced tubular cell death via the Smad3-p16-dependent G1 cell cycle arrest mechanism (24). It is also reported that quercetin can inhibit proinflammatory cytokine expression in a cisplatin-induced mouse model of AKI by suppressing Mincle/Syk/NF-κB signaling (31). However, it remains unknown whether treatment with quercetin inhibits COVID-19 AKI via the Mincle-dependent mechanism, which was also investigated in the present study *in vivo* and *in vitro*.

## Materials and methods

### Preparation of SARS-CoV-2 N protein-expressing plasmid

Mammalian expression plasmids for pcDNA3.1(+)-Flag-N were constructed and synthesized by GenScript (Nanjing, China) and GenBank accession number is MW617760.1. The pcDNA3.1(+)-Flag-N and pcDNA3.1(+)-Empty-Vector (EV) were purified by EndoFree Maxi Plasmid Kit (DP117, TIANGEN BIOTECH, Beijing, China). The primers used in this study were as follows:

Flag-N: Forward: GCGGATCCATGTCTGATAATGGACCCCA;

Reverse: GCTCTAGATTAGGCCTGAGTTGAGTCAG.

### Mouse model of AKI and treatment with quercetin

A mouse model of AKI was induced in the male db/db mice at the age of 16 weeks by ultrasound-microbubble-mediated kidney-specifically transferring SARS-CoV-2 N protein-expressing plasmid as previously described (24–26). Quercetin was dissolved in 2% DMSO and then mixed with jelly in the mouse food. Groups of 6 db/db mice at the age of 16 weeks were given oral quercetin at dosages of 150mg/kg/day from day 0 before ultrasound-microbubble-mediated SARS-CoV-2 N gene transfer until being killed on day 2 as previously described (24). In the present study, there were 5 groups of db/db mice, including untreated, EV (empty vector), NP (SARS-CoV-2 N protein), NP +QUE (NP+quercetin), and NP+DMSO (NP+DMSO-control). A group of 6 db/m mice at the age of 16 weeks was used as normal control.

### Cell lines and cell cultures

The mouse tubular epithelial cells (mTEC) were a gift from Dr. Jeffrey B. Kopp (National Institutes of Health) and were transfected with SARS-CoV-2 N protein expressing plasmid as previously described (24–26). The mTEC with overexpressing SARS-CoV-2 N (or empty vector)-expressing plasmid were cultured in DMEM/F12 medium (11320082, Gibco, ThermoFisher) supplemented with 10% FBS, 1%(v/v) penicillin-streptomycin (P/S) (15070063, Gibco, ThermoFisher). Cells were stimulated with or without advanced glycation end products (AGE, 100μg/ml, ab51995, Abcam), a hyperglycemia-related products associated with the development of diabetic kidney disease for 48 hours to obtain HMGB1-rich conditional medium for the macrophage activation studies as described below.

A mouse macrophage cell line RAW264.7 was purchased from the American Type Culture Collection (ATCC) (Manassas, VA). RAW264.7 were cultured in DMEM (11965118, Gibco, ThermoFisher) supplemented with 10% FBS, 1%(v/v) P/S. To study the inhibitory effect of quercetin on M1 macrophage activation and proinflammatory cytokine production. RAW264.7 were pretreated with quercetin (32μM) or 0.05% DMSO for 24 hours prior to the addition of HMGB1-rich conditional medium. At least three independent experiments were performed in each study.

### Small interfering RNA transfection

To knock down Mincle, RAW264.7 cells were transfected with small interfering RNA against mouse Mincle (sense 5’-CCUUUGAACUGGAAACAUUTT-3’, antisense 5’-AAUGUUUCCAGUUCAAAGGTT-3’) (designed and synthesized by Shanghai GenePharma Co., Ltd., China) by using Lipofectamine™ RNAiMAX (13778150, Invitrogen, Thermo Fisher Scientific) according to the manufacturer’s instructions. A scramble sense control was used as negative control (NC). At least three independent experiments were performed in each study.

### Immunohistochemistry

Immunohistochemistry was performed on paraffin-embedded tissue sections (3μm) using endogenous horseradish peroxidase blocking and microwave-based antigen retrieval technique if necessary (32). The antibodies used in this study included Ultra-LEAF purified anti-mouse F4/80 (123164, Biolegend) and p-p65(ab97726, Abcam). After incubation with the primary antibody overnight at 4°C, sections were incubated with anti-rabbit EnVision+ System-HRP Labelled Polymer (K4003, DAKO) at room temperature for 60 min. Then color was developed with a diaminobenzidine tablet (045-22833, FUJIFILM Wako Pure Chemical Corporation) and the nuclei were counterstained with Hematoxylin (H-3404, Burlingame) if necessary. The stained sections were viewed under a LEICA CRT6000 Light Microscope. The positive cells were counted in 6 random areas of kidney sections under the power field (x20) of a microscope and expected as positive rate or cells/mm^2^.

### Multiplex immunofluorescence

To detect the co-localization between Mincle and F4/80 expression, paraffin-embedded kidney sections (3μm) were blocked with endogenous horseradish peroxidase and then incubated with Ultra-LEAF purified anti-mouse antibody against F4/80 (123164, Biolegend) overnight, followed by adding rabbit polyclonal antibody to Mincle (BS-8541r, Bioss) as previously described (24–26). The fluorescence was developed using the Alexa Fluor ™ 488 Tyramide Reagent (B40953, Invitrogen) or Alex Fluor ™ 568 Tyramide Reagent (B40956, Invitrogen), and the nuclei were counterstained with Hoechst 33342 (H1399, Invitrogen) according to the manufacturer’s protocol. All staining sections were detected and photographed by a ZEISS AXIO Microscope. The positive co-location cells were counted in 6 random areas of kidney sections under the high-power field (x40) of a microscope and expected as positive cells/mm^2^.

### Enzyme-linked immunosorbent assay

The serum from mouse and the supernatant from cultured mTEC were collected and the concentrations of HMGB1 were measured with a mouse HMGB1 ELISA Kit according to the manufacturer’s instructions (E-EL-M0676C, Elabscience).

### Flow cytometry

Total kidney tissues were digested by Liberase TM Research Grade (Roche, Indianapolis, IN) and RAW264.7 cells were digested with 0.25% trypsin–EDTA into cell suspension. All cells were fixed by IC Fixation Buffer (00-8222-49, Invitrogene) for 10 minutes. Then cells were co-stained with CD68-PE antibody (137014, Biolegend), Mincle-FITC antibody (bs-8541R-FITC, Bioss), iNOS-APC antibody (17-5920-82, eBioscience), or CD206-FITC antibody (141704, Biolegend) overnight at 4°C. After being labeled, cells were gated and analyzed by using a BD LSRFortessa flow cytometer (BD Biosciences), and the data were analyzed by FlowJo software (V10).

### Western blot analysis

The total protein of mouse kidney tissues and RAW264.7 cells were extracted by RIPA lysis buffer (P0013B, Beyotime, Shanghai, China). The proteins were electrophoresed in a 10% SDS-PAGE gel and transferred to a Nitrocellulose Transfer Membrane (66485, Pall Corporation, Mexico). Then the membranes were blocked for 1 h at room temperature with 5% skim milk or BSA and incubated with primary antibodies at 4°C overnight. The next day, the membranes were incubated with mouse IgG (H&L) antibody DyLight™ 800 conjugated (610-145-002, ROCKLAND) or rabbit IgG (H&L) antibody DyLight™ 800 conjugated (611-145-002, ROCKLAND). The expression levels of protein were visualized by the Odyssey Infrared Imaging System (San Diego, CA, USA) and quantitatively analyzed by the Image J software (NIH, Bethesda, MD, USA). The antibodies used in this study included mouse antibodies against Mincle (sc-390806, Santa Cruz), TLR-4(sc-293072, Santa Cruz), β-actin (sc-69879, Santa Cruz) and rabbit antibodies against HMGB1(ab79823, Abcam), p-Syk (#2710, Cell Signaling Technology), Syk (#13198, Cell Signaling Technology), p-p65(ab86299, Abcam), p65(#8242, Cell Signaling Technology), iNOS (ab15323, Abcam).

### Real-time PCR assay

Total RNA from mice kidneys and RAW264.7 cells were extracted with TRIzol reagent (TR118, Molecular Research Center) following the manufacturer’s instructions. Real-time quantitative-PCR was measured with QuantStudio™ 6 and 7 Flex Real-time PCR systems (4489826, ThermoFisher) and SYBR Green Supermix (1725122, Bio-Rad). The primers used in this study included mouse Mincle: forward 5’-CCAAGTGCTCTCCTGGACGATA-3’, reverse 5’-CTGATGCCTCACTGTAGCAGGA-3’; mouse TNF-a: forward 5’- CATCTTCTCAAAATTCGAGTGACAA-3’, reverse 5’- TGGGAGTAGACAAGGTACAA CCC-3’; mouse IL-6: forward 5’- GTCCTTCCTACCCCAATTTCCA-3’, reverse 5’-TAAC GCACTAGGTTTGCCGA-3’; mouse MCP-1:forward 5’-TTAAAAACCTGG ATCGG AA C CAA-3’, reverse 5’-GCATTAGCTTCAGATTTACGGGT-3’; mouse IL-4:forward 5’-C C C CAGCTAGTTGTCATCCT-3’, reverse 5’- TGGTGTTCTTCGTTGCTGTG-3’; mouse IL-10: forward 5’-CGGGAAGACAATAACTGCACCC-3’ reverse 5’-CGGTTAG CAG TATG TTG TCCAGC-3’; mouse β-actin: forward 5’-GTGACGTTGACA T CCGTAAAGA-3’, reverse 5’-GCCGGACTCATCGTACTCC-3’.

### Statistical analyses

All data were presented as the mean ± SD. Statistical analysis was performed with one-way ANOVA for multiple groups from GraphPad Prism 9.0 Software (GraphPad, San Diego, CA, USA). P values less than 0.05 were considered statistically significant.

## Results

### Overexpression of SARS-CoV-2 N protein exacerbates renal inflammation in diabetic kidney of db/db mice, which is associated with increased HMGB1 and Mincle-expressing M1 macrophage infiltration

**Figure 1:**
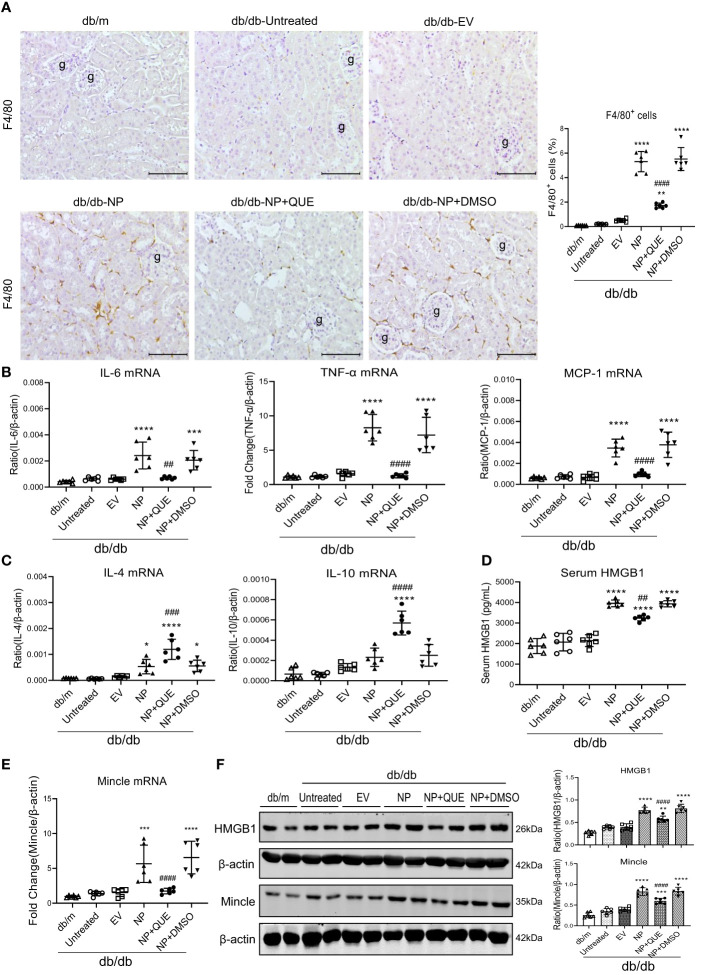
Kidney-specifically overexpressing SARS-CoV-2 N protein promotes renal inflammation in db/db mice at the age of 16 weeks by enhancing the release of HMGB1 and expression of Mincle, which is inhibited by treatment with quercetin. **(A)** Immunohistochemistry for F4/80^+^ macrophages infiltrating the kidney of db/db mice treated with or without quercetin. **(B, C)** Real-time PCR for proinflammatory cytokines (IL-6, TNF-α and MCP-1) and anti-inflammatory cytokines (IL-4 and IL-10) mRNA expression in the diabetic kidney treated with or without quercetin. **(D)** Serum levels of HMGB1. **(E)** Real-time PCR for renal Mincle mRNA expression. **(F)** Western blot analysis for expression of HMGB1 and Mincle in the kidney of db/db mice treated with or without quercetin. Each dot represents one mouse and data are expressed as the mean ± SD for groups of 6 mice. * p<0.05, **p<0.01, ***p<0.001, **** p<0.0001 versus empty vector control group (db/db-EV); ##p<0.01, ### p<0.001, #### p<0.0001 versus DMSO-treated control group(db/db-NP+DMSO). g, glomerulus; scale bar=100μm.

**Figure 2:**
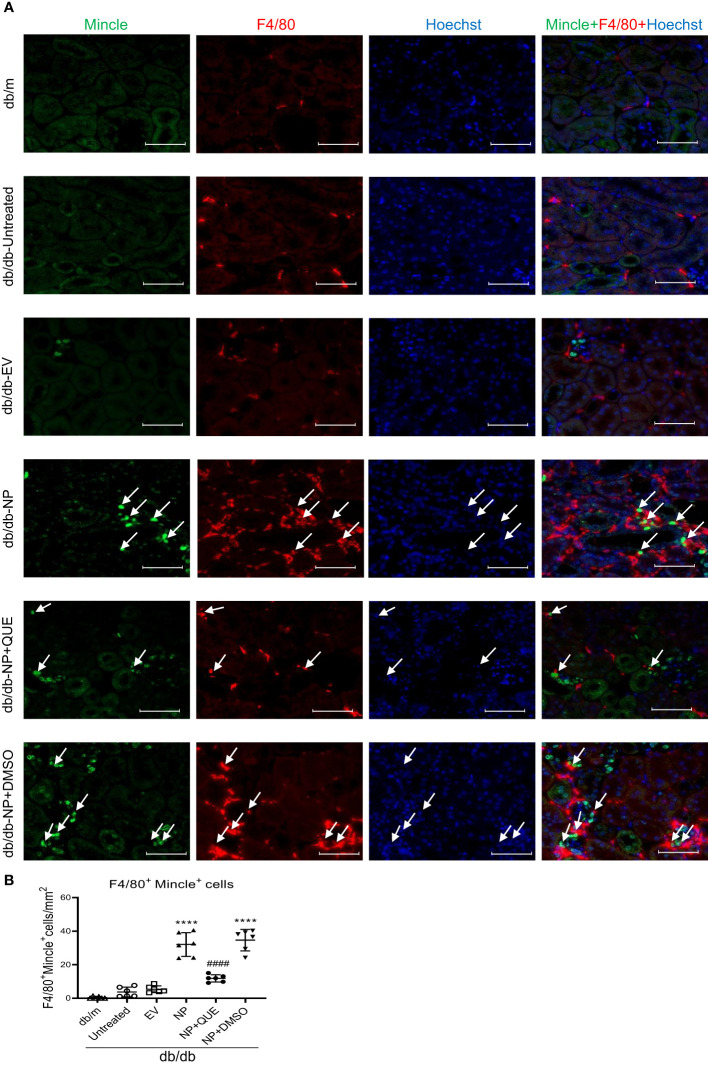
Tow-color immunofluorescence detects that overexpression of SARS-CoV-2 N protein triggers Mincle-expressing macrophages infiltrating the AKI kidney at the age of 16-week db/db mice, which is inhibited by quercetin treatment. **(A)** Two-color immunofluorescence shows the co-localization of Mincle (green) and F4/80(red) macrophages infiltrating the kidney of db/db mice treatment with or without quercetin. **(B)** Semi-quantitative analysis of Mincle-expressing macrophages (F4/80^+^Mincle^+^). Each dot represents one mouse and data are expressed as the mean ± SD for groups of 6 mice. **** p<0.0001 versus empty vector control group (db/db-EV); #### p<0.0001 versus DMSO-treated control group(db/db-NP+DMSO). Scale bar=50μm.

**Figure 3:**
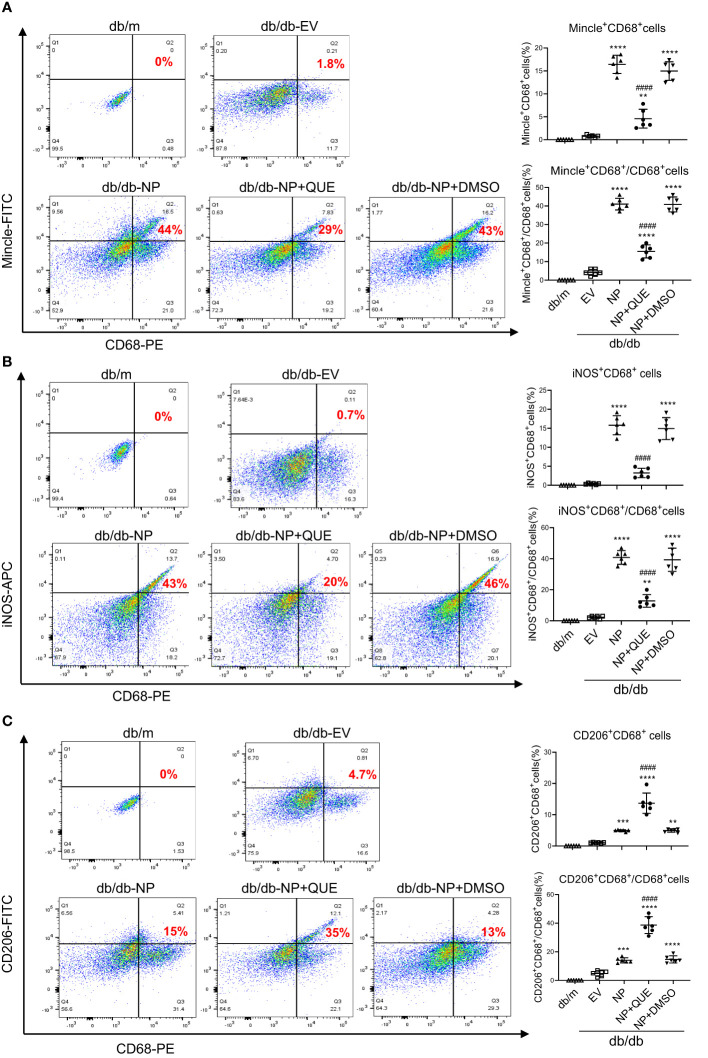
Two-color flow cytometry detects that overexpression of SARS-CoV-2 N protein largely promotes M1 macrophages infiltrating the AKI kidney at the age of 16-week db/db mice, which is inhibited by quercetin treatment. **(A)** Mincle^+^ cluster of CD68^+^ macrophages identified by Mincle^+^CD68^+^ cells in the AKI kidney of db/db mice treated with or without quercetin. **(B)** M1 macrophages identified by iNOS^+^CD68^+^ cells. **(C)** M2 macrophages identified by CD206^+^CD68^+^ cells. Note that quercetin treatment inhibits the Mincle-expressing macrophages and switches the M1 macrophages to M2 phenotype in SARS-CoV-2 N protein-induced AKI kidney of db/db mice. Each dot represents one mouse and data are expressed as the mean ± SD for groups of 6 mice. **p<0.01, ***p<0.001, **** p<0.0001 versus empty vector control group (db/db-EV); #### p<0.0001 versus DMSO-treated control group(db/db-NP+DMSO).

As inflammation is a feature of COVID-19 patients with AKI (7–17, 33). To explore the pathological link between renal inflammation and COVID-19 AKI, we first examined renal inflammation in the AKI kidney of 16-week-old db/db mice induced by overexpressing SARS-CoV-2 N protein. We found that ultrasound-microbubble-mediated overexpression of SARS-CoV-2 N protein greatly enhanced F4/80^+^ macrophage accumulation and expression of proinflammatory cytokines such as IL-6, TNF-α and MCP-1 (**Figures 1A, B**). This was associated with an increase in both serum and renal tissue levels of HMGB1, one of the DAMP molecules, and upregulation of Mincle at both mRNA and protein levels (**Figures 1D-F**). Further studies by two-color immunofluorescence and flow cytometry clearly detected that overexpression of SARS-CoV-2 N protein largely promoted M1 proinflammatory macrophages infiltrating the kidney by co-overexpressing F4/80 and Mincle/iNOS markers, which was largely increased in the diabetic kidney of db/db mice (**Figures 2**, **3A, B**). These observations suggest that overexpression of SARS-CoV-2 N protein may mediate severe AKI under diabetic conditions by triggering the release of DAMP molecules such as HMGB1 from the necrotic tubular cells, which then may activate M1 macrophages and stimulate the production of proinflammatory cytokines to exacerbate further AKI via a Mincle-dependent mechanism.

### Overexpression of SARS-CoV-2 N protein promotes M1 pro-inflammatory macrophage activation and renal inflammation under diabetic conditions by activating Mincle-Syk-NF-κB signaling

**Figure 4:**
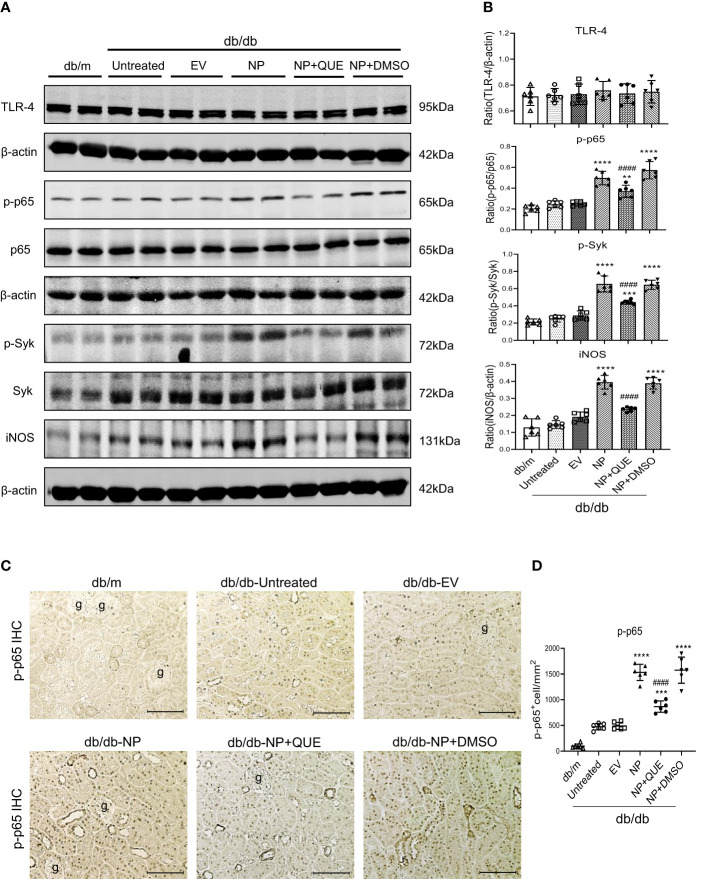
Kidney-specifically overexpressing SARS-CoV-2 N protein activates Syk/NF-κB signaling in db/db mice at the age of 16 weeks, which is inhibited by treatment with quercetin. **(A, B)** Western blot analysis of activation of Syk (p-Syk), NF-κB(p-p65) and expression of TLR-4 and iNOS in the kidney of db/db mice treated with or without quercetin. **(C, D)** Immunohistochemistry for detecting activation of NF-κB (p-p65 nuclear translocation). Note that treatment with quercetin inhibits phosphorylation of Syk and NF-κB as well p65 nucleated translocation in SARS-CoV-2 N protein-induced AKI kidney. Each dot represents one mouse and data are expressed as the mean ± SD for groups of 6 mice. ** p<0.01, *** p<0.001, **** p<0.0001 versus empty vector control group (db/db-EV); #### p<0.0001 versus DMSO-treated control group(db/db-NP+DMSO). g, glomerulus; scale bar=100μm.

It is well known that Mincle is a typical PRR expressed by M1 proinflammatory macrophages and can recognize the endogenous DAMPs released by necrotic cells. The binding of DAMPs to Mincle can activate Syk and NF-κB signaling by phosphorylation (19). Our previous study also demonstrated that LPS induces M1 macrophage activation in AKI via Mincle/Syk/NF-κB-dependent mechanism (34). In the present study, western blotting and immunohistochemical staining also detected that overexpressing SARS-CoV-2 N protein caused severe renal inflammation with massive M1 macrophage infiltration in the diabetic kidney of db/db mice, which was associated with upregulation of Mincle on macrophages (**Figures 2**, **3A, B**) and activation of Syk/NF-κB signaling (**Figure 4**).

**Figure 5:**
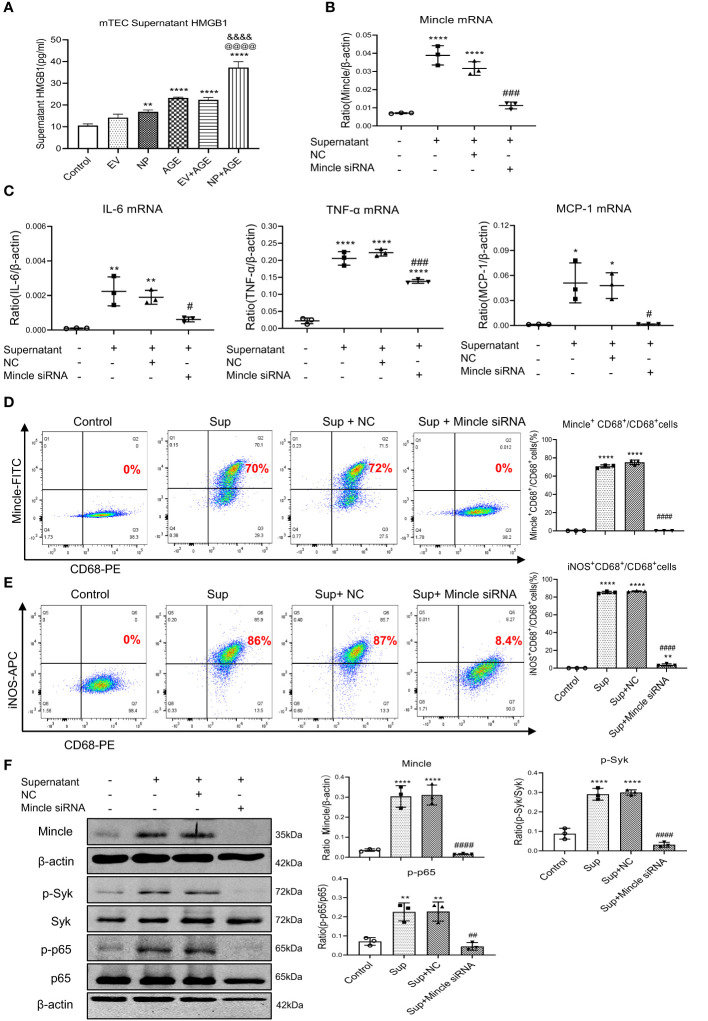
Overexpression of SARS-CoV-2 N protein in mouse tubular epithelial cells (mTEC) largely promotes the release of HMGB1 to activate M1 macrophages in RAW264.7 cells via the Mincle-dependent mechanism *in vitro*. **(A)** Concentrations of HMGB1 in the supernatant of dead mTEC induced by overexpressing SARS-CoV-2 N protein in the presence or absence of AGE (100µg/ml for 48 hours). **(B, C)** Real-time PCR shows that silencing macrophage Mincle blocks HMGB1-contained mTEC supernatant (Sup)induced upregulation of Mincle, IL-6, TNF-α, and MCP-1 mRNA expression in RAW264.7 cells. **(D, E)** Flow-cytometry analysis reveals that silencing macrophage Mincle blocks HMGB1-contained mTEC supernatant (Sup) induced M1 macrophages as determined by Mincle^+^CD68^+^ and iNOS^+^CD68^+^ populations in RAW264.7 cells. **(F)** Western blot analysis shows that silencing macrophage Mincle blocks HMGB1-contained mTEC supernatant (Sup) induced activation of Mincle-Syk/NF-κB signaling in RAW264.7 cells. Each dot represents one experiment and data are presented as mean ± SD for at least three independent experiments. *p<0.05, ** p<0.01, **** p<0.0001 versus control group; @@@@ p<0.0001 versus AGE group; &&&& p<0.0001versus Flag-empty vector with AGE group (EV+AGE); # p<0.05, ## p<0.01, ### p<0.001, #### p<0.0001 versus cells treated with supernatant from SARS-CoV-2 N protein-induced dead mTEC and negative control siRNA (Sup+NC).

To further confirm the necessary role for Mincle in M1 macrophage-mediated AKI in response to SARS-CoV-2 N protein under diabetic conditions, we performed serial studies in SARS-CoV-2 N protein-overexpressing tubular cells under high AGEs conditions. We found that either SARS-CoV-2 N protein or AGEs were capable of inducing equal levels of HMGB1 released from injured tubular cells, indicating that either SARS-CoV-2 N protein or AGEs can induce tubular cell injury to release HMGB1. Interestingly, once SARS-CoV-2 N protein-overexpressing tubular cells were cultured with AGEs, the release of a DAMP molecule HMGB1 became double (**Figure 5A**), suggesting that SARS-CoV-2 N protein can potentiate HMGB1 released from injured tubular cells under diabetic conditions. To further determine whether SARS-CoV-2 N protein can activate M1 macrophages via the Mincle-dependent mechanism, we cultured macrophages (RAW264.7) with high HMGB1-contained supernatant obtained from AGEs-stimulated SARS-CoV-2 N protein-expressing tubular cells. As expected, the addition of high HMGB1-contained supernatant largely promoted Mincle expression by macrophages, resulting in a marked activation of M1 proinflammatory macrophages by co-expressing CD68 and Mincle/iNOS and production of IL-6, TNF-α, and MCP-1 (**Figures 5B-E**). All these changes were blocked by specifically silencing macrophage Mincle with siRNA (**Figures 5B-E**), demonstrating that SARS-CoV-2 N protein may trigger M1 macrophage activation and proinflammatory response via the Mincle-dependent mechanism. This was further confirmed by western blotting that silencing macrophage Mincle suppressed HMGB1-induced Mincle expression and activation of Syk/NF-κB signaling (**Figure 5F**).

### Treatment with quercetin attenuates SARS-CoV-2N protein-induced AKI in diabetic db/db mice by blocking Mincle-mediated-M1 macrophage activation via a Syk-NF-κB-dependent mechanism *in vivo*

Our recent study showed that quercetin can effectively block SARS-CoV-2 N protein-induced tubular cell death by targeting the Smad3-p16-dependent G1 cell cycle arrest mechanism (24). In the present study, we further investigated whether treatment with quercetin can attenuate SARS-CoV-2 N protein-induced AKI in diabetic db/db mice by blocking M1 macrophage activation and renal inflammation in diabetic db/db mice via a Mincle-dependent mechanism. As shown in **Figure 1**, treatment with quercetin largely inhibited SARS-CoV-2 N protein-induced F4/80^+^ macrophages infiltrating the diabetic kidney (**Figure 1A**) and greatly suppressed the mRNA expression of IL-6, TNF-α, and MCP-1 while increasing the expression of IL-4 and IL-10 mRNA levels (**Figure 1B, C**). This was associated with the inhibition of both serum and renal tissue levels of HMGB1 and expression of Mincle in the diabetic kidney of db/db mice (**Figures 1D-F**), suggesting that treatment with quercetin may inhibit the release of DAMPs such as HMGB1 from necrotic renal tubular cells and thus suppresses M1 macrophage activation and renal inflammation. This was further demonstrated by two-color immunofluorescence, demonstrating that treatment with quercetin inhibited Mincle-expressing F4/80^+^ macrophage infiltrating the diabetic kidney of SARS-CoV-2 N protein-induced AKI (**Figure 2**). Further studies by two-color flow cytometry also confirmed this notion that treatment with quercetin significantly inhibited SARS-CoV-2 N protein-induced M1 macrophages by co-expressing Mincle^+^CD68^+^and iNOS^+^CD68^+^ macrophages while increasing CD206^+^CD68^+^ macrophages (**Figure 3**). These findings suggest that quercetin may result in the switching of macrophage properties from M1 to M2 macrophage phenotype.

As Mincle is a typical PRR expressed by macrophages and can recognize endogenous DAMPs such as HMGB1 released by the necrotic cells to activate the downstream Syk/NF-κB signaling (19, 34), we further examined whether treatment with quercetin inhibits SARS-CoV-2 N protein-induced M1 macrophage activation and renal inflammation via the Mincle-Syk/NF-κB signaling pathway. Interestingly, although treatment with quercetin did not alter the expression of TLR-4 (**Figures 4A, B**), it did significantly suppress Mincle expression (**Figure 1F**) and therefore inhibited phosphorylation of Syk and NF-κB/p65 in the diabetic kidney of SARS-CoV-2 N protein-induced AKI (**Figure 4**).

### Quercetin inhibits SARS-CoV-2 N protein-induced M1 macrophage activation while promoting M2 macrophages via Mincle-dependent Syk/NF-κB signaling in RAW264.7 cells

**Figure 6:**
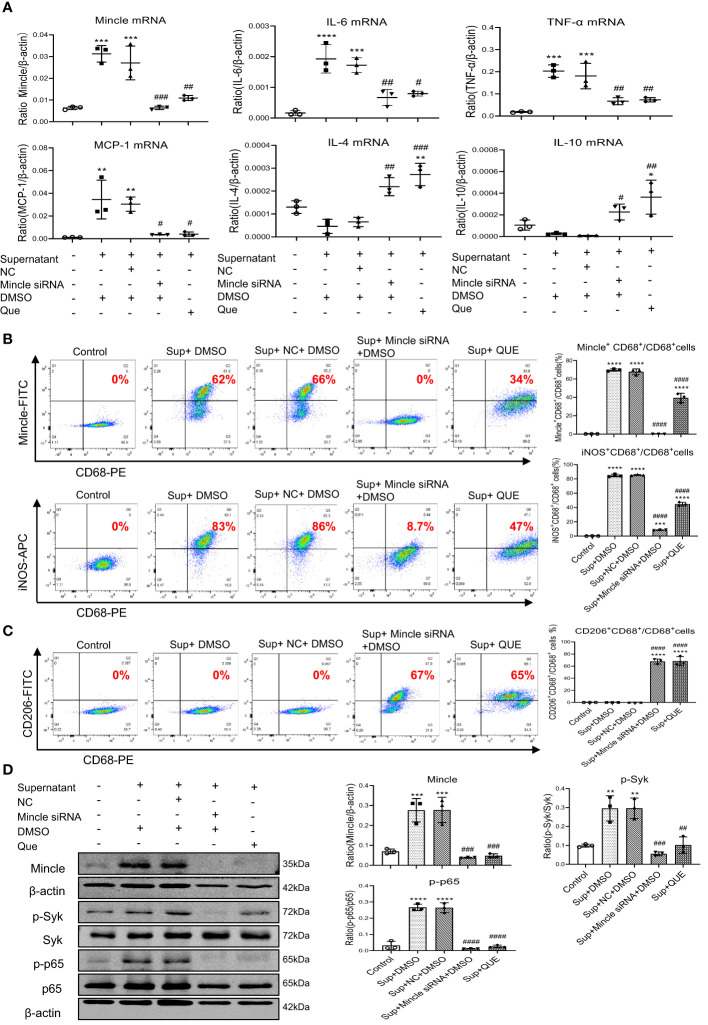
Quercetin inhibits HMGB1-contained mTEC supernatant-induced M1 macrophages while promoting M2 populations by targeting Mincle-Syk/NF-κB signaling in RAW264.7 cells. **(A)** Real-time PCR detects that like a Mincle siRNA, pre-treatment of RAW264.7 cells with quercetin (32µM) for 24 hour is able to inhibit Mincle, IL-6, TNF-α, MCP-1, while increasing IL-4 and IL-10 mRNA expression induced by the HMGB1-contained supernatant (Sup) from SARS-CoV-2 N protein-expressing mTEC. **(B, C)** Two-color flow-cytometry analysis reveals that like specifically silencing macrophage Mincle, pre-treatment with quercetin (32µM) for 24 hour inhibits M1 (Mincle^+^CD68^+^ and iNOS^+^CD68^+^) while increasing M2 (CD206^+^CD68^+^) macrophage populations induced by the HMGB1-contained supernatant (Sup) from SARS-CoV-2 N protein-expressing mTEC. **(D)** Western blot analysis shows that like treatment with Mincle siRNA, pre-treatment with quercetin blocks Mincle-Syk/NF-κB signaling in RAW264.7 cells in response to the HMGB1-contained supernatant (Sup) from SARS-CoV-2 N protein-expressing mTEC. Each dot represents one experiment and data are presented as mean ± SD for at least three independent experiments. * p<0.05, ** p<0.01, *** p<0.001, **** p<0.0001 versus control group; # p<0.05, ## p<0.01, ### p<0.001, #### p<0.0001 versus cells treated with supernatant from SARS-CoV-2 N protein-induced dead mTEC and negative control siRNA and DMSO group (Sup+NC+DMSO).

To further confirm the mechanism of quercetin in the inhibition of SARS-CoV-2 N protein-induced M1 macrophage activation, we treated RAW264.7 cells with HMGB1-contained medium obtained from SARS-CoV-2 N protein-overexpressing mouse tubular cells as described above. Mincle-dependent mechanism in M1 macrophage activation was confirmed by treating RAW264.7 cells with the Mincle siRNA. Results showed that, like Mincle siRNA, treatment with quercetin was capable of inhibiting HMGB1-induced M1 macrophage activation by suppressing the expression of Mincle and pro-inflammatory cytokines including IL-6, TNF-α, and MCP-1 while increasing anti-inflammatory cytokines such as IL-4 and IL-10 expression (**Figure 6A**). Two-color flow cytometry also revealed that the addition of quercetin resulted in the shift from M1 to M2 macrophages as demonstrated by reducing about 50% of the Mincle^+^CD68^+^ and iNOS^+^CD68^+^ M1 macrophages while increasing more than 50% of CD206^+^CD68^+^ macrophages (**Figures 6B, C**). Further study by western blot analysis also confirmed this notion that the addition of quercetin blocked the activation of Mincle-Syk-NF-κB signaling under HMGB1-rich supernatant (**Figure 6D**). Taken together, these findings suggest that quercetin inhibits SARS-CoV-2 N protein-induced AKI under diabetic conditions by switching M1 to M2 macrophage phenotype via the Mincle/Syk/NF-κB signaling.

## Discussion

Our previous studies demonstrated that ultrasound-microbubble-mediated kidney-specifically overexpressing SARS-CoV-2 N protein is capable of inducing kidney tubular necrosis and causing AKI via Smad3-dependent G1 cell cycle arrest and necroptosis mechanisms (24–26). In the present study, we identified that SARS-CoV-2 N protein caused AKI by promoting M1 macrophage activation and renal inflammation via a Mincle-dependent mechanism, which added new information to the previous findings that SARS-CoV-2 N protein can activate NLRP3 and NF-κB to induce hyperinflammation (35, 36). We found that SARS-CoV-2 N protein-induced renal tubular cell necrosis in diabetic db/db mice resulted in the release of HMGB1, a DAMP molecule that can bind Mincle on macrophages and activate M1 macrophages via Mincle-Syk/NF-κB signaling. It is now clear that HMGB1 is an abundant non-histone nuclear protein that can be secreted into the extracellular environment and serves as an essential DAMP to activate proinflammatory signaling (37). HMGB1 can activate M1 macrophages in mouse models of ischemia-reperfusion and obstruction kidney disease and *in vitro (* 38–41). In critically ill COVID-19 patients, serum HMGB1 is elevated and correlated with levels of inflammatory cytokines (7, 21, 22). The present study also found that a large amount of HMGB1 was released from injured tubular cells induced by overexpressing SARS-CoV-2 N protein under diabetic conditions *in vivo* and *in vitro*. Importantly, we also uncovered that HMGB1 could activate M1 macrophages via the Mincle-dependent mechanism, specifically silencing macrophage Mincle protected against HMGB1-induced M1 macrophage activation and production of signature cytokines such as IL-6, TNF-α, and MCP-1. It is well documented that Mincle plays an important role in renal inflammation and is a key factor for triggering and maintaining the M1 macrophage phenotype. Blockade of Mincle on macrophages can protect against cisplatin-induced AKI (34, 42). Consistent with these previous findings, we found that SARS-CoV-2 N protein-induced AKI in db/db mice was associated with a marked increase in Mincle-expressing macrophages (Mincle^+^CD68^+^) and iNOS^+^CD68^+^ M1 macrophages. It is highly possible that overexpression of SARS-CoV-2 N protein could largely promote the release of DAMPs such as HMGB1 from injured renal tubular cells in db/db mice, resulting in high levels of HMGB1 in both serum and renal tissues. After being released, HMGB1 could bind and activate Mincle on macrophages and then stimulate M1 macrophage activation and production of proinflammatory cytokines such as IL-6, TNF-α, and MCP-1 via the Syk/NF-κB pathway. This was further confirmed in RAW264.7 cells in which specifically silencing macrophage Mincle blocked HMGB1-induced activation of Mincle-Syk/NF-κB signaling and thus blocked M1 macrophage activation and proinflammatory cytokine production. It should be pointed out that HMGB1 is one of the DAMPs released from SARS-CoV-2 N protein overexpressing tubular cells and many other DAMP molecules released from the injured tubular cells in response to overexpression of SARS-CoV-2 N protein may also contribute to activate proinflammatory macrophages. Indeed, besides HMGB1 (7, 21, 22), other DAMP molecules such as S100A8/A9, SP-A, CIRBP, and histone may also participate in M1 proinflammatory macrophage activation in response to COVID-19 infection as previously reported (21). This novel finding may well explain the clinical notions that DAMPs can cause the “cytokine storm” and lead to organ damage in critically ill COVID-19 patients (17). Thus, SARS-CoV-2 N protein is pathogenic in COVID-19 AKI. It can induce tubular cell death via the Smad3-dependent G1 cell cycle arrest and necroptosis mechanisms as previously reported (24–26). It may also cause severe AKI under diabetic conditions by activating M1 macrophages and promoting massive renal inflammation via the Mincle-dependent mechanism. However, it should be pointed out that systemic inflammatory responses such as “cytokine storm” after COVID-19 infection may also contribute to the M1 macrophage activation. It has been well documented that there are excessive immune responses with massive production of proinflammatory cytokines such as IL-6, IL-1β, TNF-a, and MCP-1 in patients with severe SARS-CoV-2 infection (8–13). These proinflammatory cytokines can activate M1 macrophages systemically and then promote their migration into the diseased kidney where they become further activated and maintain the M1 proinflammatory phenotype via Mincle-dependent mechanism as previously reported (34). Nevertheless, in the present study, macrophages may be primarily activated locally within the kidney via the Mincle-dependent mechanism as AKI was induced by overexpressing SARS-CoV-2 N protein locally in the diabetic kidney.

In the present study, we also uncovered that quercetin functions as a Mincle inhibitor to block Mincle/Syk/NF-κB signaling, thereby inhibiting M1 while promoting M2 macrophage activation in SARS-CoV-2 N protein-induced AKI in db/db mice. Quercetin is a natural flavonoid compound, which is widely found in various heat-clearing and detoxifying herbs and food. Quercetin has antiviral, anti-inflammatory, antioxidant, and other biological activities (43). Many studies suggest that quercetin is effective for the treatment of patients with COVID-19. Both experimental and clinical trials showed that quercetin has a therapeutic effect on COVID-19-associated AKI (27–30, 44). *In vitro*, quercetin can inhibit LPS-induced M1 macrophages while promoting M2 macrophage differentiation (45), indicating that quercetin ameliorates renal injury in AKI by regulating macrophage polarization. Our previous study also showed that quercetin inhibits M1 while upregulating M2 macrophages by blocking Mincle/Syk/NF-κB signaling in cisplatin-induced AKI mouse models and in LPS-induced bone marrow-derived macrophages (31). Interestingly, the present study found that treatment with quercetin inhibited SARS-CoV-2 N protein-induced Mincle but not TLR4 expression in db/db mice with AKI. This suggested that Mincle but not TLR4 may be involved in the M1 macrophage activation during the development of SARS-CoV-2 N protein-induced AKI. We have previously reported that treatment with quercetin can inhibit SARS-CoV-2 N protein-induced tubular cell death via the Smad3-p16-dependent G1 cell cycle arrest mechanism (24). This may also inhibit the release of DAPMs such as HMGB1 from the injured tubular cells and inactivate M1 proinflammatory macrophages by suppressing the binding of HMGB1 to Mincle. Thus, quercetin treatment inhibited macrophage activation and progressive renal inflammation in SARS-CoV-2 N protein-induced AKI via a Mincle-dependent mechanism. This was further confirmed in cultured macrophages in which the addition of quercetin was capable of inhibiting HMGB1-induced Mincle expression and activation of Syk/NF-κB signaling, thereby blocking M1 while promoting M2 macrophage activation. Thus, consistent with previous findings clinically (27, 28), quercetin may be an effective therapeutic agent for COVID-19 AKI (27, 28). Furthermore, results from this study also revealed that blockade of Mincle-Syk/NF-κB-mediated M1 macrophage activation may be a novel molecular mechanism through which quercetin treatment improves the severity of COVID-19 patients clinically.

In summary, SARS-CoV-2 N protein is pathogenic for AKI and may mediate AKI by activating M1 macrophages via a Mincle-Syk/NF-κB-dependent mechanism. Quercetin is a therapeutic agent for SARS-CoV-2 N protein-induced AKI in db/db mice and may inhibit AKI by switching M1 to M2 macrophage activation, which may be associated with inactivation of Mincle signaling.

## Data availability statement

The original contributions presented in the study are included in the article/supplementary material. Further inquiries can be directed to the corresponding authors.

## Ethics statement

The animal study was approved by Animal Experimentation Ethics Committee at the Chinese University of Hong Kong. The study was conducted in accordance with the local legislation and institutional requirements.

## Author contributions

HL: Writing – review & editing. WWu: Writing – original draft. WWa: Investigation, Writing – original draft. LL: Writing – original draft. JC: Writing – original draft. SS: Writing – original draft. BW: Writing – original draft. YZ: Software, Writing – original draft. JL: Writing-review & editing. XH: Writing – review & editing. XW: Writing – review & editing. XY: Writing – review & editing.
